# Pharmacogenomic profiling of the South Korean population: Insights and implications for personalized medicine

**DOI:** 10.3389/fphar.2024.1476765

**Published:** 2024-12-03

**Authors:** Mi Seon Youn, Se Hwan Ahn, Ju Han Kim

**Affiliations:** ^1^ Seoul National University Biomedical Informatics (SNUBI), Department of Biomedical Sciences, Seoul National University College of Medicine, Seoul, Republic of Korea; ^2^ Seoul National University Biomedical Informatics (SNUBI), Division of Biomedical Informatics, Seoul National University College of Medicine, Seoul, Republic of Korea

**Keywords:** pharmacogenomics, pharmacogenetics, drug dosing recommendation, South Korean, PGx profiling

## Abstract

Adverse drug reactions (ADRs) pose substantial public health issues, necessitating population-specific characterization due to variations in pharmacogenes. This study delineates the pharmacogenomic (PGx) landscape of the South Korean (SKR) population, focusing on 21 core pharmacogenes. Whole genome sequencing (WGS) was conducted on 396 individuals, including 99 healthy volunteers, 95 patients with chronic diseases, 81 with colon cancer, 81 with breast cancer, and 40 with gastric cancer, to identify genotype-specific drug dosing recommendations. Our detailed analysis, utilizing high-throughput genotyping (HTG) of CYP2D6 and comparative data from the 1,000 Genomes Project (1 KG) and the US National Marrow Donor Program (NMDP), revealed significant pharmacogenetic diversity in core pharmacogenes such as CYP2B6, CYP2C19, CYP4F2, NUDT15, and CYP2D6. Notably, intermediate metabolizer frequencies for CYP2B6 in SKR (3.28%) were comparable to Europeans (5.77%) and East Asians (5.36%) but significantly differed from other global populations (*p* < 0.01). For CYP2C19, 48.74% of SKR individuals were classified as intermediate metabolizers, with the *35 allele (2.02%) being unique to SKR, the allele not observed in other East Asian populations. Additionally, the high-risk *3 allele in CYP4F2 was significantly more frequent in SKR (34.72%) than in other East Asian populations (*p* < 0.01). NUDT15 poor metabolizers were found in 0.76% of SKR, aligning closely with other East Asians (1.59%), while TPMT poor metabolizers were predominantly observed in Europeans and Africans, with one case in SKR. We identified significant allele frequency differences in CYP2D6 variants rs1065852 and rs1135840. Among the 72 drugs analyzed, 93.43% (n = 370) of patients required dosage adjustments for at least one drug, with an average of 4.5 drugs per patient. Moreover, 31.31% (n = 124) required adjustments for more than five drugs. These findings reveal the substantial pharmacogenetic diversity of the SKR population within the global population, emphasizing the urgency of integrating population-specific PGx data into clinical practice to ensure safe and effective drug therapies. This comprehensive PGx profiling in SKR not only advances personalized medicine but also holds the potential to significantly improve healthcare outcomes on a broader scale.

## 1 Introduction

Therapeutic failure and adverse drug reactions (ADRs) constitute significant public health challenges (Coleman and Pontefract, 2016; Schork, 2015). ADRs not only contribute substantially to the burden and costs associated with pharmacological treatments (Budnitz et al., 2007; Budnitz et al., 2011) but the absence of therapeutic efficacy further escalates healthcare expenditures and resource utilization. This issue pervades various disease states and drug classes (Zhdanava et al., 2020; Pilon et al., 2019a; Amos et al., 2018; Olfson et al., 2018; Pilon et al., 2019b; Pilon et al., 2020; Pan et al., 2018; Rush et al., 2006). Pharmacogenomics (PGx) emerges as a promising discipline aimed at optimizing drug selection and dosing to mitigate ADRs and enhance drug efficacy (Weinshilboum and Wang, 2017; Cecchin and Stocco, 2020). Studies have consistently shown that genetic variations affecting drug absorption, distribution, metabolism, excretion, and toxicity can substantially influence pharmacological outcomes. The variability in the frequency of PGx variants across different populations represents a primary obstacle to the widespread clinical integration of PGx (Al-Mahayri et al., 2020), highlighting the critical need for population-specific PGx profiling (Bachtiar and Lee, 2013; Bonifaz-Peña et al., 2014; Jittikoon et al., 2016; Wilson et al., 2001).

Advancements in next-generation sequencing (NGS) technologies have introduced various approaches for PGx profiling. Many studies have adopted whole exome sequencing (WES) or targeted NGS panels for large-scale PGx assessments (Al-Mahayri et al., 2020; Gordon et al., 2016), though these strategies are constrained by their inability to analyze non-coding regions (Londin et al., 2014). Whole Genome Sequencing (WGS) offers the most comprehensive approach for PGx profiling, overcoming these limitations (Londin et al., 2014).

The US Food and Drug Administration (FDA) has recognized PGx markers on over 500 medications (Center for Drug Evaluation and Research, 2023). Instruments such as the Pharmacogenomics KnowledgeBase (PharmGKB) (Whirl-Carrillo et al., 2012) and guidelines from the Clinical Pharmacogenetics Implementation Consortium (CPIC) (Caudle et al., 2014; Caudle et al., 2014) play a pivotal role in facilitating the clinical application of PGx tests for distinct drug-gene interactions, backed by solid evidence. PharmGKB and CPIC employ star-allele nomenclature system (Robarge et al., 2007). However, Prioritization in the clinical implementation of the drug-gene pair differs depending on populations (Mette et al., 2012; Yasuda et al., 2008; Sirugo et al., 2019). For example, CPIC suggests employing population-specific dosing guidelines based on star-alleles for drugs such as warfarin (Johnson et al., 2017). While there are similarities in physical features, culture, and lifestyles among East Asians including Han Chinese and Japanese, genomic differences indicate that South Koreans should be considered as an independent population (Wang et al., 2018).

The Korean Variant Archive 2 (KOVA2) comprises a vast genome repository of the Korean population, encompassing 1896 WGS and 3409 WES datasets, totaling 5,305 individuals (Lee J. et al., 2022). However, there is currently a shortage of PGx insights derived from this archive. For instance, genotyping highly polymorphic pharmacogenes like CYP2D6 is challenging due to the limitations of WGS. In contrast, our study provides a detailed PGx analysis of the South Korean (SKR) population using the HTG method for CYP2D6, offering an additional method compared to previous studies.

This study aims to enhance therapeutic outcomes by providing clinicians with novel insights into the PGx diversity of the SKR population, emphasizing the importance of integrating population-specific PGx markers into clinical practices for more personalized and effective healthcare.

## 2 Materials and methods

### 2.1 Study cohort

**FIGURE 1:**
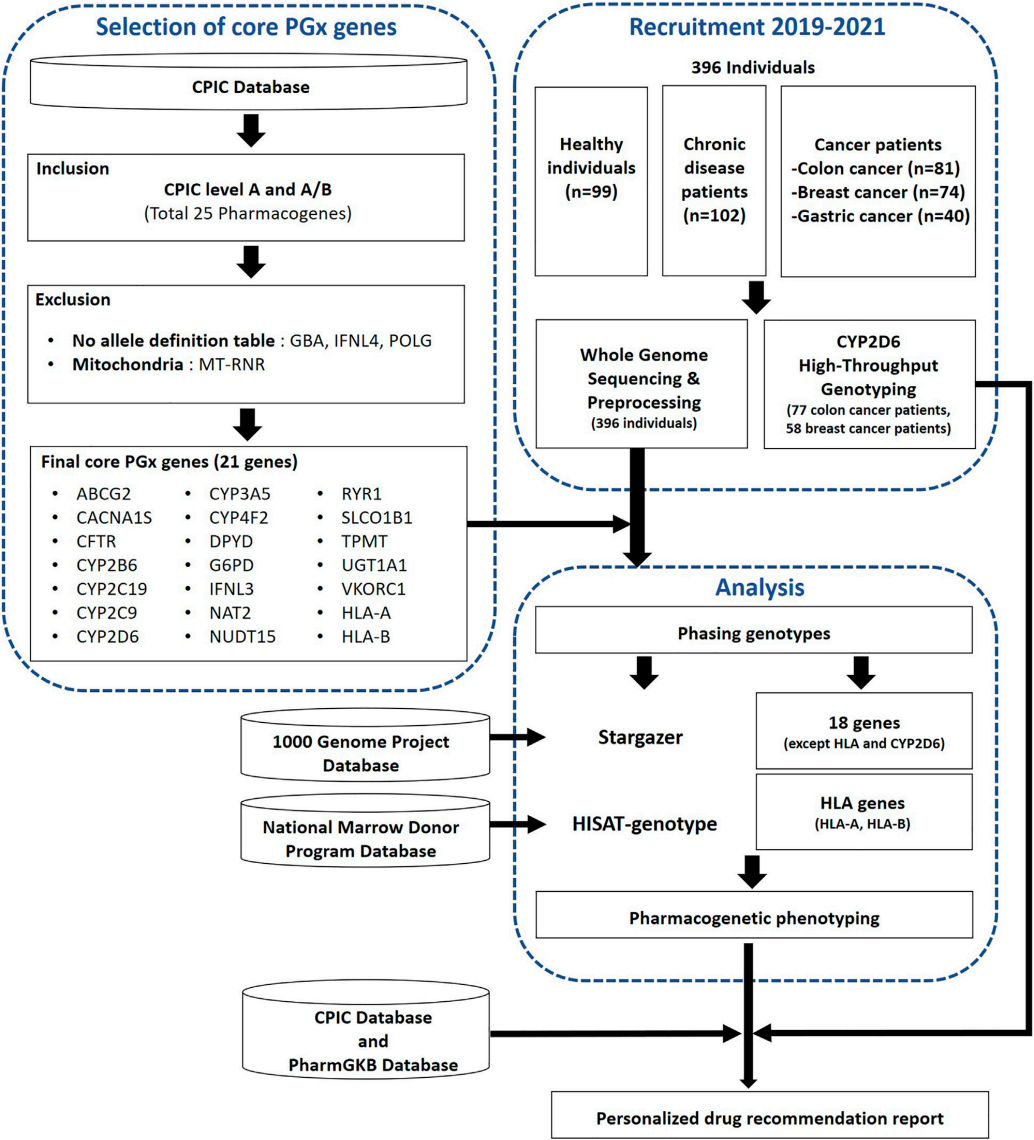
A workflow of the selection process for core pharmacogenes and the subsequent analysis to determine pharmacogenomic (PGx) characteristics in the study population. The analysis encompassed whole genome sequencing (WGS) for 396 individuals, complemented by CYP2D6 high-throughput genotyping (HTG) performed on a subset of 135 individuals from this group. Additionally, the diagram indicates the practicality of utilizing PGx data to generate personalized medication recommendation reports for this population.

In this study, conducted between 2019 and 2021, we systematically recruited each of the five participant groups, comprising both healthy individuals and four distinct disease groups (chronic diseases, colon cancer, breast cancer, gastric cancer), from five separate medical institutions. Ethical approval for this study was granted by the Institutional Review Board of each group with all donors provided written informed consent if available (SKKU 2020-03-019-001, 2019-0909-011, 2003-119-1110, 3-2020-0257, B-2006-621-303). From these participants, we collected blood samples for the production of genomic data. We prospectively collected blood samples from a cohort of 356 unrelated SKR individuals, including healthy individuals, chronic disease patients, and colon and breast cancer patients, and retrospectively collected samples from 40 unrelated SKR individuals (Figure 1). The SKR cohort was collected as part of “The Korean Healthcare Bigdata showcase Project” through the Korea Health Industry Development Institute (KHIDI). In assembling the cohort, our primary objective was to collect a diverse sample for future studies, including healthy individuals, patients with various chronic diseases, and those with three different types of cancer.

### 2.2 Whole genome sequencing (WGS) and quality control and variant calling

DNA was extracted from Whole Blood sample using Exgene blood SV mini kit (GeneAll) according to the manufacturer’s protocol. Briefly, 200 µL Blood Sample add 20 µL proteinase K, 200 µL. (Optional: add 20 µL of RNase solution (20 mg/mL) to the sample, pipet 2∼3 times to mix and incubate for 2 min at room temperature.) Add buffer BL in a 1.5 mL microcentrifuge tube and incubate at 56°C for 10 min. The lysate was 200 µL ethanol (96%–100%) with vortexing. For the binding of DNA to the spin column, sample was transferred with 700 µL. Then according to standard operating procedures, after the binding of DNA to the spin column, residual contaminants were washed away by buffers BW, TW. Finally, DNA was eluted in 50 ∼ 100 µL buffer AE.

The quality and quantity of purified DNA were assessed by fluorometry (Qubit, Invitrogen) and gel electrophoresis. Briefly, 100 ng of genomic DNA from each sample were fragmented by acoustic shearing on a Qsonica 800 R2 instrument. Fragments of 350 bp were ligated to Illumina’s adapters and PCR-amplified. 500–600 bp is appropriate size for final library. Libraries were quantified using the TapeStation 4,200 instrument (Agilent Technologies) and KAPA Library Quantification Kit (KK4824, Kapa Biosystems).

The resulting purified libraries were applied to an Illumina flow cell for cluster generation and sequenced using 150 bp paired-end reads on an Illumina NovaSeq 6,000 (Illumina) sequencer by following the manufacturer’s protocols.

The genomic analysis method proceeded as follows: Initially, the Trimmomatic (v.0.36) (Bolger et al., 2014) was utilized to eliminate adapter sequences and filter out low-quality reads, where reads with over 10% N bases or less than Q20 base quality score in more than 40% of the read were discarded. Subsequently, the filtered sequences were aligned to the reference genome (hg19) using the BWA (v.0.7.17) (Li and Durbin, 2010) with a minimum seed length of 45. Duplication reads resulting from PCR were then eliminated using the GATK (Depristo et al., 2011) MarkDuplicates (v.4.0.11.0). Following this, the GATK (Depristo et al., 2011) Base Recalibrator (v.4.0.11.0) corrected misaligned base quality scores for sequences with removed duplication reads, and the GATK (Depristo et al., 2011) Haplotype Caller (v.4.0.11.0) was employed for variant calling. Annotations for identified variants were subsequently added using the SnpEff (v.4.3t) (Cingolani et al., 2012).

### 2.3 High-throughput genotyping (HTG) for CYP2D6 gene

For genotyping, DNA concentration was normalized to 50 ng/μL, with A260/280 and A260/230 ratios between 1.5 and 2, verified by the QuantiFluor^®^ dsDNA system (Promega) and NanoDrop ND-2000 Spectrophotometer (Biotek). We employed plates with 48 subarrays, each featuring 64 through-holes, for 192 SNVs across 16 samples, using VIC^®^ and FAM^®^ fluorophore-labeled probes. Each DNA sample, 2.5 µL, was mixed with an equal volume of TaqMan^®^ OpenArray™ Master Mix in a 384-well plate, and loaded using the OpenArray™ AccuFill^®^ system (Applied Biosystems). Sealed in glass boxes from the OpenArray™ kits, the plates underwent a 4-h amplification in the QuantStudio 12K Flex Real-time PCR System (Applied Biosystems), with analysis conducted using TaqMan^®^ Genotyper software (Life Technologies).

### 2.4 Selection of core pharmacogenes

From 25 initial candidates, we identified 21 core pharmacogenes following CPIC Level A and A/B guidelines. Both GBA and POLG genes were excluded due to missing allele definition tables of CPIC guidelines, and IFNL4 was omitted as a polymorphic pseudogene. MT-RNR were also excluded, the former being a mitochondrial gene. The finalized list comprises ABCG2, CYP2B6, CACNA1S, CFTR, CYP2C19, CYP2C9, CYP2D6, CYP3A5, CYP4F2, DPYD, G6PD, IFNL3, NAT2, NUDT15, RYR1, SLCO1B1, TPMT, UGT1A1, VKORC1, HLA-A, and HLA-B.

### 2.5 Allele assignment and phenotype prediction

To compile a comprehensive PGx haplotyping compendium, we employed two tools for variant calling in core pharmacogenes. Stargazer (version 1.0.8) (Lee et al., 2019) was utilized to assign star-alleles and predict molecular phenotypes in 18 core pharmacogenes, excluding CYP2D6 and HLA genes. For the high polymorphism in HLA genes, HLA-A and HLA-B genotyping in 395 WGS datasets was performed using HISAT-genotypes (version 1.3.2) (Kim et al., 2019), excluding one sample due to inadequate data depth in the HLA region. HISAT-genotype algorithm facilitates HLA typing and DNA fingerprinting from standard WGS data.

### 2.6 Comparison of SKR cohort with global populations

We compared allele and phenotype frequencies of 19 core pharmacogenes, excluding HLA genes, across various populations using data from the 1,000 Genomes Project (1 KG) (Auton et al., 2015). To account for the significant polymorphism among populations, we subdivided groups that were genetically and geographically similar to our SKR population. Specifically, the East Asian (EAS) Ancestry group was segmented into Southern Han Chinese (CHS), Japanese (JPT), Han Chinese (CHB), Kinh Vietnamese (KHV), and Dai Chinese (CDX) for more precise comparisons. Our analysis involved using a Fisher-exact test to compare star-allele and phenotype frequencies in our cohort of 396 SKR individuals with the global data from the 1 KG. Additionally, allele frequencies of HLA-A and HLA-B loci in SKR population were compared with those from eight other populations, using data from the US National Marrow Donor Program (NMDP) (Gragert et al., 2013). To represent our study population and facilitate these comparisons, we conducted a principal component analysis (PCA) of HLA gene allele frequencies, using MATLAB version 19b for all analyses.

### 2.7 Analysis of the distribution of drugs associated with pharmacogenes

We analyzed prescription recommendations as per CPIC guidelines for medications requiring dose adjustment or discontinuation. We defined the drug categories based on required recommendations: “Standard” (no change), “Up” (increased dose), “Down” (decreased dose), “Alternative” (recommend against use, with alternative suggestions), and “Consider implications” (requires further consideration) (Supplementary Table S4). Volatile anesthetic agents, including desflurane and sevoflurane; proton pump inhibitors (PPIs), such as omeprazole and pantoprazole; and non-steroidal anti-inflammatory drugs (NSAIDs), like celecoxib and ibuprofen, were systematically grouped. Special categorizations were applied to drugs necessitating dosage modifications for pediatric patients. For drug phenotypes labeled as “Indeterminate”, which signify ambiguous drug recommendations, a conventional approach was adopted, with outcomes detailed accordingly. Notably, G6PD was associated with 35 drugs requiring prescription adjustments according to the 2022 CPIC guidelines (Gammal et al., 2023). Warfarin, necessitating a dose control algorithm (Johnson et al., 2017), was omitted. Thiopurine medications—mercaptopurine, azathioprine, and thioguanine—require gene-specific dosage adjustments (Relling et al., 2019). Phenytoin dosing was evaluated based on CYP2C9 alleles, using the CPIC diplotype-phenotype table (Karnes et al., 2021), with a focus on loading doses. For voriconazole and clopidogrel, adult-specific guidelines were applied (Lee C. R. et al., 2022; Moriyama et al., 2017).

## 3 Results

### 3.1 Star-allele distributions by populations

**FIGURE 2:**
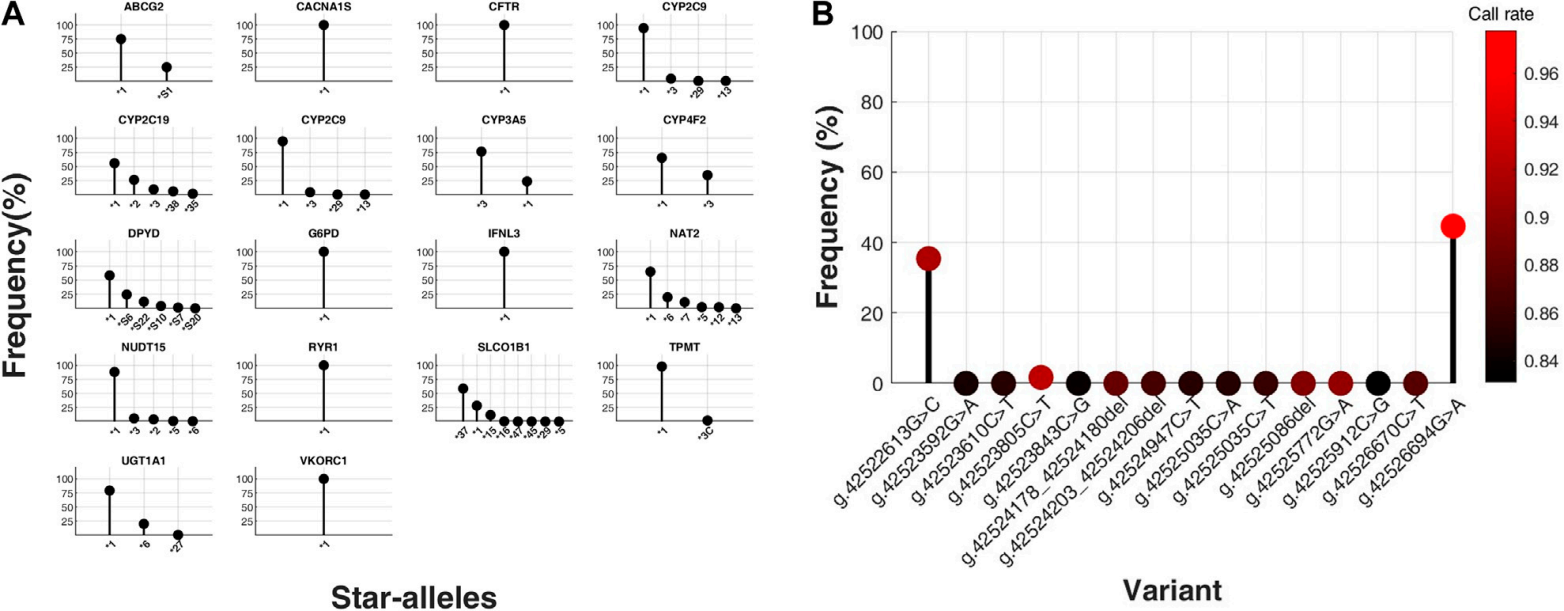
Distribution of star-alleles and variants in core pharmacogenes, excluding HLA genes, from a total of 21. **(A)** Star-allele frequencies within core pharmacogenes, excluding CYP2D6 and HLA genes, in a cohort of 396 South Korean (SKR) individuals, presented as percentages. The *x*-axis categorizes the identified star-alleles for each gene, whereas the *y*-axis quantifies their respective frequencies as percentages within the SKR group (n = 396). **(B)** Frequencies of variants for CYP2D6 gene were determined in a population of 135 individuals from South Korea, and the results were expressed as percentages. The *x*-axis represents the observed types of variants for the CYP2D6 gene, while the *y*-axis represents the corresponding percentages in SKR population (n = 135). Dot colors represent variant call rates, ranging from black (0.83) to red (0.98).

The overall workflow of this study includes the selection of core pharmacogenes and pharmacogenetic analyses such as genotyping and phenotyping (Figure 1). We conducted an analysis of star-allele distributions among core pharmacogenes, excluding CYP2D6 and HLA genes, within a cohort of 396 SKR individuals. Figure 2A illustrates the frequencies of distinct star-alleles across these core pharmacogenes. Notably, genes such as CACNA1S, CFTR, G6PD, IFNL3, RYR1, and VKORC1 exhibited a dominant star-allele frequency of 100% as reference star-alleles, indicating a high level of genetic uniformity within the SKR population for these specific genes. In contrast, genes like CYP2B6, CYP2C9, and NUDT15 displayed a broader spectrum of star-alleles with substantially lower frequencies, suggesting the presence of genetic diversity within the population. DPYD, NAT2, and SLCO1B1, in particular, demonstrated diverse star-allele distributions; DPYD featured six alleles, and SLCO1B1 had eight, highlighting a wide range of PGx variability.

HTG via the OpenArray platform was performed on 135 participants, targeting 15 CYP2D6 variants, including frame shift deletion, intron, and missense mutation types. The genotyping data exhibited an average call rate of 93.00%, with the highest call rate at 97.79% and the lowest at 83.09%. Analysis revealed that, with the exception of three out of the 15 variants, all participants displayed the wild type genotype for the remaining variants (Figure 2B).

We conducted a comparative analysis of star-allele frequencies between the SKR population and global populations from the 1 KG, highlighting the distinct genetic landscapes across core pharmacogenes, excluding CYP2D6 and HLA genes (Supplementary Figure S1; Supplementary Table S5).

**FIGURE 3:**
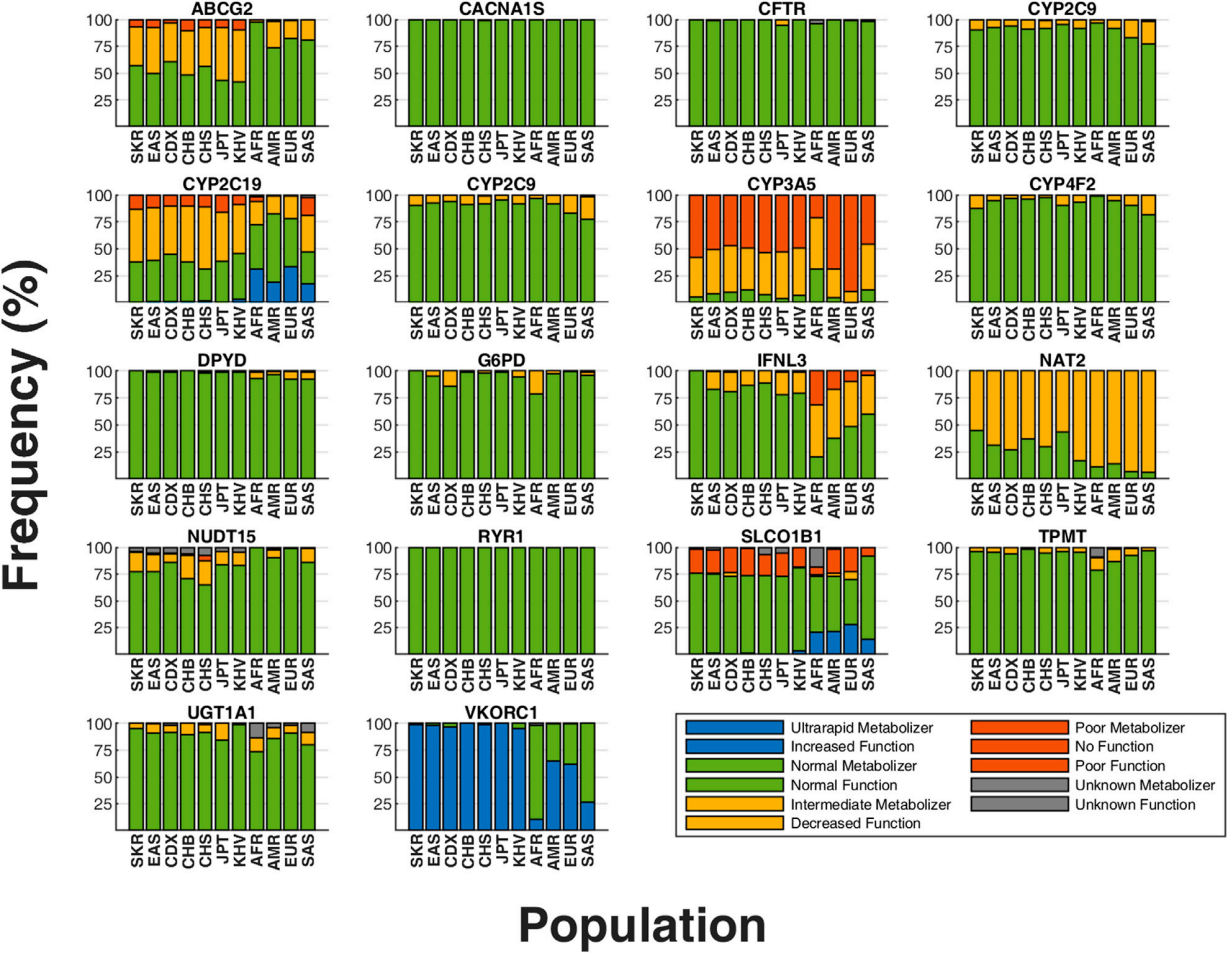
Comparison of phenotype frequencies between SKR population and 1,000 Genomes Project (1 KG) populations. Distribution of predicted phenotypes for core pharmacogenes, excluding CYP2D6 and HLA genes. The *x*-axis represents individual populations, while the *y*-axis shows the proportion of predicted phenotypes for each gene. Unique colors correspond to each distinct predicted phenotype in these graphs. SKR, South Korean (our study cohort); AFR, Africans; AMR, Admixed Americans; SAS, South Asians; EUR, Europeans; EAS, East Asians; CHS, Southern Han Chinese; JPT, Japanese; CHB, Han Chinese; KHV, Kinh Vietnamese; CDX, Dai Chinese.

Phenotypes derived from these star-alleles reveal substantial frequency variances between the SKR and 1 KG populations, including EAS subgroups (Figure 3). SKR exhibited the closest distribution to the EAS among major populations. However, significant PGx differences exist among EAS, notably in CYP2B6, CYP2C19, CYP4F2, NUDT15, and TPMT where SKR displayed distinct distribution patterns compared to other populations (see Supplementary Tables S6–S8).

The frequency of intermediate metabolizers of the CYP2B6 gene in SKR (3.28%) was similar to that of Europeans (EUR) (5.77%) compared to EAS (5.36%), but significantly different from other major populations such as Admixed Americans (AMR) (17.86%), South Asians (SAS) (15.13%), and Africans (AFR) (27.84%) (*p* < 0.01) (Supplementary Table S8). According to the phenotype definitions provided by stargazer (Sahana et al., 2022), the *9/*9 genotype was classified as an intermediate metabolizer (3.28% in SKR), aligning closely with EAS frequencies (5.36%), but with notable difference observed among subpopulation within EAS. Although there was a slight variance in frequency compared to geographically proximate populations like the CHB (2.91%) and JPT (7.69%), SKR exhibited considerably lower frequencies than CDX (13.98%) despite belonging to the same EAS (*p* < 0.01) (Supplementary Table S7). Additionally, ultrarapid metabolizers were observed in all major populations except for SKR.

In the analysis of CYP2C19 gene, 48.74% of SKR population were identified as intermediate metabolizers, a proportion similar to EAS (48.81%) but significantly higher than AMR (16.43%). For the poor metabolizer phenotype, the frequency in SKR (13.64%) is slightly higher than in EAS (11.71%) and CHB (10.68%), but lower than in JPT (16.35%). Notably, the *2 allele of CYP2C19 shows a lower frequency in SKR (26.39%) compared to other EAS populations (31.25%), indicating substantial inter-population genetic diversity. Moreover, the *35 allele, not observed in other EAS groups, is present in the SKR population at a frequency of 2.02%, emphasizing unique genetic traits within the SKR cohort. Certain drugs should be avoided by CYP2C19 phenotypes, including intermediate metabolizers, likely poor metabolizers, and poor metabolizers (Lee C. R. et al., 2022; Moriyama et al., 2017; Bousman et al., 2023; Hicks et al., 2017; Lima et al., 2021). The analysis of diplotypes among intermediate and poor metabolizers, including specific percentages, highlights the complex genetic variations influencing drug metabolism and reinforces the need for personalized pharmacogenetic approaches in clinical settings.

The prevalence of the high-risk *3 allele in the CYP4F2 gene, associated with decreased function (Johnson et al., 2017), varies significantly across EAS populations. Specifically, the prevalence in SKR (34.72%) is significantly higher than in JPT (23.08%), CHB (21.84%), and EAS which is 21.43% (*p* < 0.01) (Supplementary Table S6).

NUDT15 poor metabolizer phenotype is most prevalent among EAS (Moriyama et al., 2016). Our study found that more than 92% of individuals from populations other than EAS and SKR carried the wild-type allele of NUDT15 gene. The poor metabolizer phenotype was identified only in EAS (1.59%), AMR (0.58%), and SKR (0.76%). As previously noted, NUDT15 poor metabolizers are predominantly found in EAS, including SKR. Furthermore, TPMT poor metabolizer phenotype is frequently identified among EUR and AFR (Relling et al., 2019). In our research, most individuals with intermediate and poor metabolizer phenotypes were from the AFR population. Additionally, we detected a case of the poor metabolizer phenotype in one sample, which is SKR. This highlights the unique genetic profile of the SKR, emphasizing the importance of population-specific PGx analysis.

The genotyping of 15 variants within the CYP2D6 gene through HTG limited the scope of phenotypic interpretation due to the restricted variant selection. This HTG data underscored significant inter-population variations in the allele frequencies of CYP2D6 gene variants, thereby highlighting the importance of population-specific pharmacogenetic diversity (Supplementary Figure S2; Supplementary Table S1). Notably, the intron variant g.42523805C>T (rs28371725) showed genetic diversity across regions in EAS (3.77%), contrasting with its prevalence in SKR (1.68%). In CHB (3.40%), the variant frequency was higher than in SKR, but is was significantly lower in JPT (0.48%), highlighting the genetic heterogeneity among regional cohorts. Moreover, the missense mutation variants g.42526694G>A (rs1065852) and g.42522613G>C (rs1135840) exhibited higher allele frequencies within SKR. The rs1065852 variant had the highest frequency in EAS (57.14%) among global populations. Within EAS subpopulations geographically close to SKR, the highest frequencies were observed in CHB (60.19%), followed by SKR (44.64%) and JPT (36.06%). Similarly, the rs1135840 variant was most prevalent in EAS (64.01%), with the highest frequencies in CHB (71.26%), SKR (35.42%), and JPT (34.18%). Particularly, the CYP2D6 enzyme is pivotal in the hydroxylation and demethylation processes of drugs with clinical importance (Gardiner and Begg, 2006). In SKR population, the CYP2D6 gene variant rs1065852 was identified as the predominant allele, as illustrated in Figure 2B.

### 3.2 HLA allele frequencies: comparative study between SKR and others using the NMDP database

**FIGURE 4:**
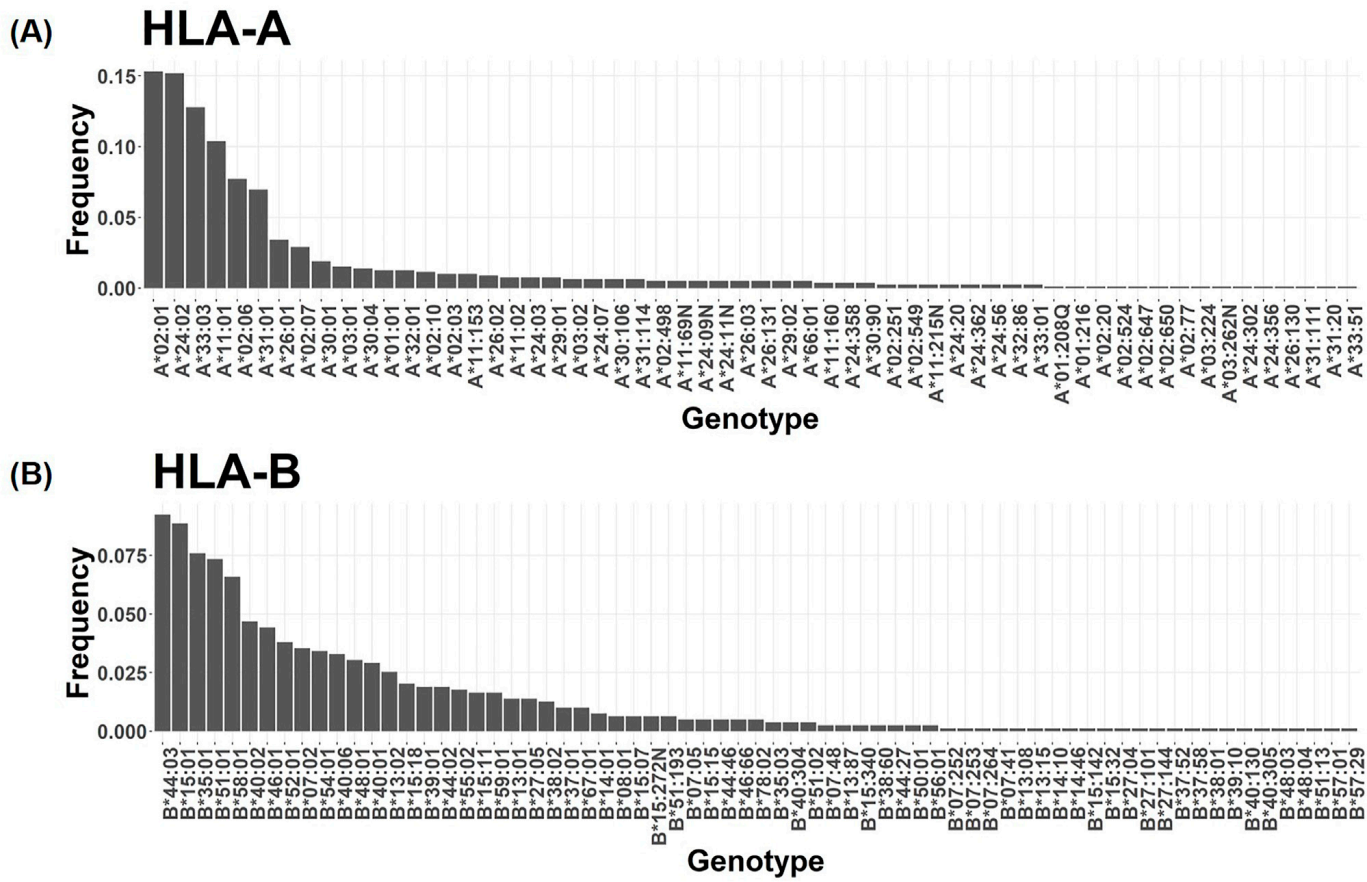
Frequency distribution of HLA-A and HLA-B in the SKR population. The genotypes are sorted in descending order based on their frequencies, with the highest frequency genotypes positioned on the left side of the graph for **(A)** the HLA-A gene and **(B)** the HLA-B gene.

Due to the high polymorphism in HLA genes, we applied HISAT-genotypes (Kim et al., 2019) for HLA-A and HLA-B genotyping across 395 SKR WGS datasets, excluding one sample for inadequate HLA region coverage. In our analysis of 395 SKR individuals, we assessed the frequencies of HLA-A and HLA-B star-alleles (Figure 4; Supplementary Tables S2, S9). Among these, six HLA-A and five HLA-B star-alleles showed frequencies above 5%, with four HLA-A alleles surpassing the 10% mark. Our study also identified 42 HLA-A and 44 HLA-B star-alleles with frequencies below 1%. The most frequent diplotypes were A*31:01:A*33:03 (7.59%) for HLA-A and B35:01:B35:01 (4.56%) for HLA-B (Supplementary Table S10).

HLA-A*02:01 was notably prevalent in SKR (15.32%). Its frequency was comparable in Korean (KORI) (18.57%) and Japanese (JAPI) (14.80%) populations (Gragert et al., 2013). However, it was less frequent in Chinese (NCHI) (9.46%), despite all being EAS. Contrastingly, this allele appeared at much lower frequencies in other Asian groups, South Asian (AINDI) (4.92%) and Other Southeast Asian (SCSEAI) (5.78%). Additionally, A*24:02 (15.19%), A*33:03 (12.79%), and A*11:01 (10.38%) each exhibited frequencies exceeding 10% in the SKR. A*24:02 in JAPI (35.30%) and A*11:01 in NCHI (27.51) both exhibited notably higher frequencies compared to SKR. In the HLA-B locus, B*44:03 was the most prevalent in SKR (9.24%), with KORI showing a similar frequency (8.50%). The allele frequency in JAPI was slightly lower (6.05%), and lower in NCHI (1.41%).

**FIGURE 5:**
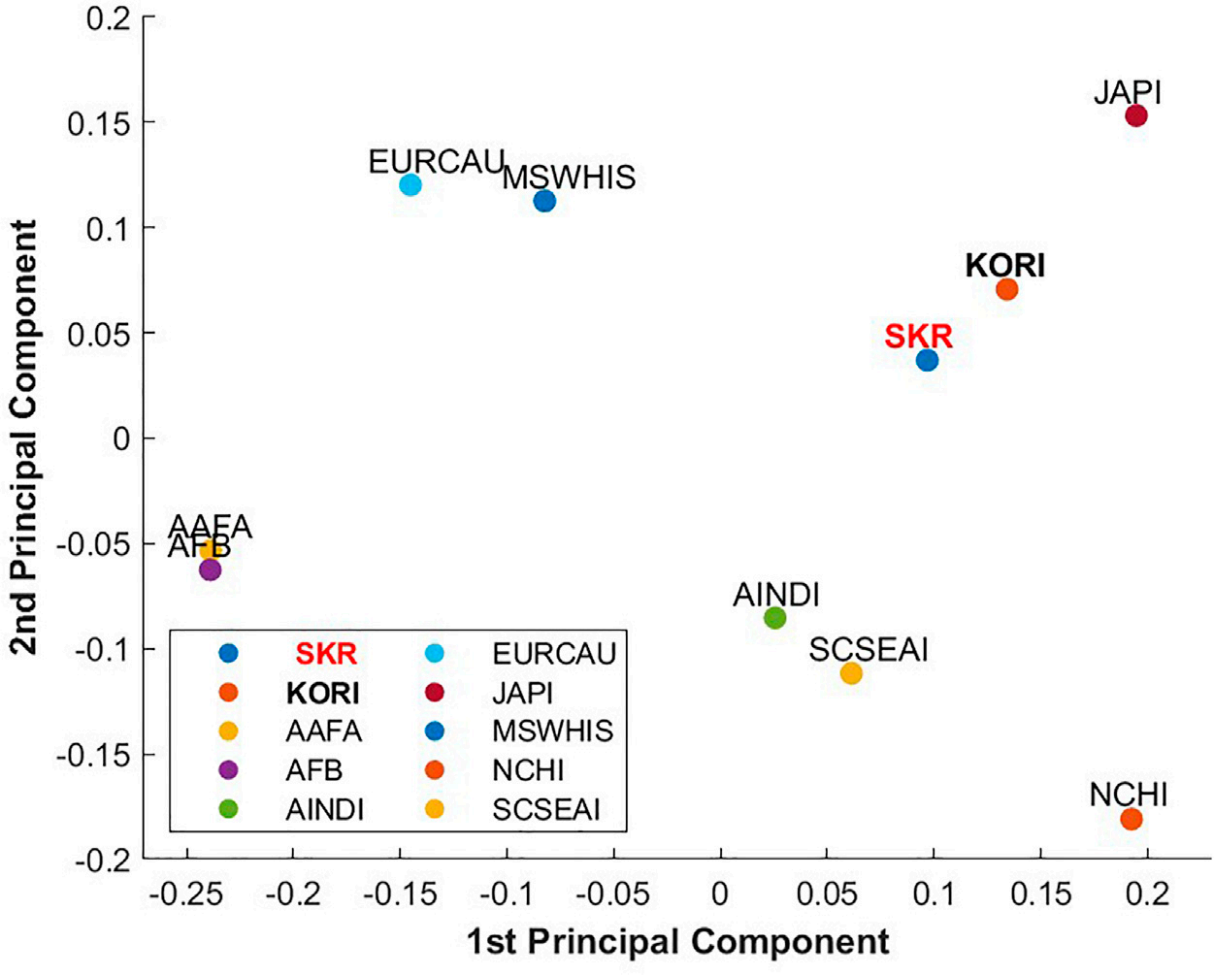
PCA Plot of HLA-A and HLA-B Star-Alleles Between SKR and NMDP Populations. This plot displays the first and second principal components comparing our study population (SKR, South Koreans living in South Korea) with clusters from the National Marrow Donor Program (NMDP) populations. SKR, South Korean (our study population); KORI, Korean Reference; JAPI, Japanese; NCHI, Chinese; AAFA, African American; AFB, African; AINDI, South Asian Indian; EURCAU, European Caucasian; SCSEAI, Other Southeast Asian; MSWHIS, Mexican or Chicano.

PCA was utilized to explore star-allele frequency variation across nine populations, including our study cohort (Figure 5). The analysis revealed that the first three principal components accounted for 45.03%, 21.72%, and 16.10% of the total frequency variation, respectively. The first principal component significantly differentiated populations by continent, whereas the second principal component distinctly separated EAS populations. Importantly, the first and second principal components showed a close genetic affinity between SKR and the NMDP SCSEAI and JAPI populations. Our findings demonstrate a notable concordance with the KORI, reinforcing the alignment of our study outcomes with existing benchmarks. Although classified under the EAS, our comparative analysis revealed differences with the JAPI and NCHI populations, highlighting the necessity for population-specific PGx profiling.

### 3.3 Analysis of the distribution of phenotypes and drugs associated with pharmacogenes

**FIGURE 6:**
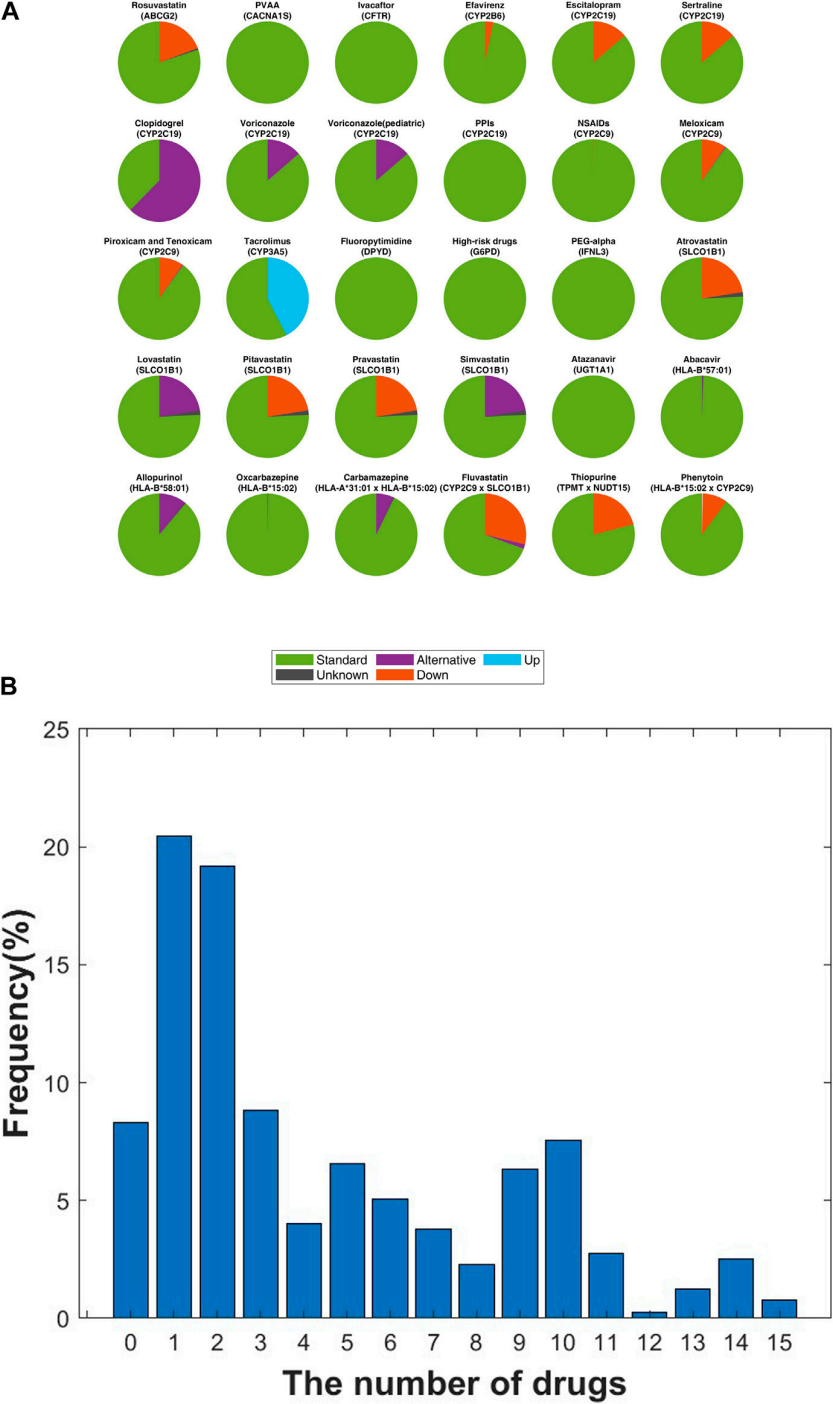
Analysis of Personalized Medication Dosing Recommendations in the SKR Population. **(A)** Within a SKR cohort of 396 individuals, the distribution of drug dosing recommendations is categorized as “Standard” (standard dose), “Down” (reduced dose), “Alternative” (advising against use, with alternative options provided), “Up” (increased dose), and “Unknown” (indeterminate dosing for certain phenotypes). **(B)** Distribution of recommended dosing modifications for each individual following CPIC guidelines. The *x*-axis represents the count of medications necessitating dosing modifications for each individual, while the *y*-axis denotes the frequency corresponding to each quantity of medications with recommended adjustments, presented as a percentage.

In this study, prescription recommendations for drugs linked to 21 core pharmacogenes were evaluated, adhering to Level A and A/B guidelines and focusing on phenotype-based directives. Figure 6A displays a comprehensive comparison of all relevant drug-gene pairs, excluding the CYP2D6 gene, with categorization based on drug actions and corresponding guidelines.

Clopidogrel, an antiplatelet medication critical for managing heart disease risk, necessitated significant prescription adjustments. A notable 62.4% (n = 247) of the SKR population exhibited a CYP2C19 molecular phenotype contraindicating its use in both cardiovascular and neurovascular contexts (Coukell and Markham, 1997). Enfuvirtide, utilized in AIDS treatment, required dosage modifications in 34.8% (n = 138) of individuals, deviating from standard guidelines, with 3.2% (n = 13) needing a reduced dose of 200 mg/day (Chen et al., 2002). Tacrolimus, essential for preventing post-transplant immune rejection, demanded increased doses beyond the standard regimen for 42.4% (n = 168) of participants (Hooks, 1994). Within the statin category, aimed at lowering LDL cholesterol to manage hyperlipidemia, lovastatin and simvastatin were deemed unsuitable for 22.5% (n = 90) of the study population, indicating a need for alternative lipid-lowering strategies (Nissen et al., 2005). HLA-B specific drugs such as abacavir and allopurinol (Martin et al., 2012; Hershfield et al., 2013) showed that 10.9% (n = 43) of carriers of HLA-B*58:01 should avoid allopurinol, while one individual (0.3%) was recommended to discontinue abacavir due to HLA-B*57:01 carriage.

Our analysis included drugs influenced by multiple genes (Figure 6A; Supplementary Table S3). A total of 72 drugs were analyzed, revealing that 93.43% (n = 370) of patients required adjustment for at least one drug, averaging 4.5 drugs per patient (Figure 6B). Notably, 31.31% (n = 124) of patients needed adjustments for more than five drugs, up to a maximum of 15 drugs.

## 4 Discussion

The evolution of personalized medicine significantly alters the paradigm of drug prescription and administration, highlighting the essential role of genetic testing in pinpointing individual metabolic variations for medications (Franceschini et al., 2018). Our research employs WGS and HTG techniques to map the PGx landscape of 20 critical pharmacogenes, including HLA genes and CYP2D6 gene, in SKR population (Jia and Zhao, 2014). The focus on CYP2D6 gene, known for its high polymorphism, necessitated this specific HTG approach, as WGS alone was insufficient for accurate genotyping of highly polymorphic genes. To address this issue, we employed targeted HTG for 15 specific variants within CYP2D6, thereby ensuring more precise genotyping for genes with high polymorphism. Some limitations of WGS, for example, repetitive regions where WGS will not map uniquely, accessing to high GC content regions, resolution of complex regions of the genome (e.g., HLA), and detecting structural variation and large segmental duplications, can be solved by utilizing long-read sequencing (Pollard et al., 2018).

We emphasize the importance of genetic testing for precise drug dosage adjustments to mitigate elevated adverse effect risks. Although our findings provide a summarized assessment of these risks, it is crucial to independently evaluate drug efficacy and adverse effect risks, highlighting the necessity for further studies to explore the exact impact of these genetic variants on drug responses. Moreover, our analysis reveals distinct PGx traits within SKR compared to other EAS groups, such as CHB and JPT (Figure 3). Notable findings include the unique prevalence of certain star-alleles like CYP2C19*3 and CYP2C19*35, which are associated with particular metabolizer phenotypes, suggesting specific drug dosages should be carefully considered to avoid adverse outcomes. The comparative rarity of the CYP2B6*9 within SKR implies that most SKR might tolerate standard doses of Efavirenz (Desta et al., 2019), a critical consideration for HIV treatment protocols. Our comparative analysis of star-allele frequencies among EAS and other populations, supported by the HLA PCA plot (Figure 5), confirms the accuracy of our PGx profiling as validated by the KORI. This underscores the importance of conducting population-specific PGx research to accurately adjust drug therapies to the unique genetic makeup of the SKR.

The comprehensive analysis not only offers groundbreaking insights into the PGx distinctions of the SKR but also sets a new precedent for future research aimed at enhancing personalized medicine strategies. Through the examination of genotype distributions and phenotype-related drug recommendations, we discovered that a substantial segment of SKR requires adjusted drug prescriptions aligned with their distinct genetic profiles. Specifically, among the 396 participants in our study, 370 individuals (93.43%) were identified as needing one or more drug modifications (Figure 6B). This research represents a notable advancement in personalized healthcare, emphasizing the need for further investigation into the specific PGx profiles of SKR and their implications for personalized medicine. We can leverage the population-specific PGx information of the SKR population to make personalized PGx reports. In our study, most individuals were recommended adjusted prescriptions, which can aid medical personnel or patients in decision-making. Consequentially, we anticipate that this approach will streamline the treatment process and enhance cost-effectiveness. Looking forward, future research could benefit from advancements in sequencing technologies, including the long-read sequencing and cost-effective strategies. These advancements hold potential for enhancing genotyping accuracy in high-polymorphic pharmacogenes such as CYP2D6, thus facilitating deeper exploration and comprehension of PGx.

This study has several limitations. First, we secured WGS data from a total of 396 South Korean individuals, despite challenges related to privacy protection regulations, difficulties in obtaining consent, and the high cost of data production. To more accurately represent the South Korean population, a larger database is needed, which remains a priority for future research. Second, we did not integrate other pharmacogenomics (PGx) databases such as PharmGKB and Dutch Pharmacogenetics Working Group (DPWG). These databases differ from CPIC in how they define phenotypes based on genotypes, making data integration challenging. However, the genes classified as CPIC Level A and A/B are supported by high or moderately high levels of evidence in PharmGKB and DPWG (Alshabeeb et al., 2022). In future studies, we plan to incorporate data from PharmGKB, CPIC, and DPWG for more comprehensive analyses. The lack of integration in this study is a limitation we aim to address in future work. Third, while rare variants are currently recognized for explaining the missing heritability in drug response phenotypes, we considered this a complex topic that requires a separate research design. As a result, incorporating rare variant analysis into this study was challenging. Given the scope of this study, rare variant analysis requires a more comprehensive approach, which we believe is an important direction for future research (Kozyra et al., 2017; Ingelman-Sundberg et al., 2018; Lauschke and Ingelman-Sundberg, 2016; Silgado-Guzmán et al., 2022).

In conclusion, our study represents a pioneering effort in PGx profiling within the SKR population using WGS, demonstrating accurate genotyping in highly polymorphic genes like CYP2D6 using HTG method. Moreover, our comparison analysis with global populations and within EAS subgroups highlighted significant differences, emphasizing the importance of comprehensive population-specific analyses in PGx research.

## Data Availability

The data analyzed in this study was obtained from “The Korean Healthcare Big Data Showcase Project” through the Korea Health Industry Development Institute (KHIDI), funded by the Korean Disease Control and Prevention Agency in the Republic of Korea (no. 4800-4848-501). The following licenses/restrictions apply: The data is planned for deposition into the Clinical & Omics Data Archive (CODA), managed by the Korea Disease Control and Prevention Agency. Public release of the data is prohibited under the terms of the consent form agreed upon by the study participants. Access to the data is restricted and subject to approval by the Institutional Review Board, limited to research determined to serve the public interest. Requests to access these datasets should be directed to MSY, yyh252090@gmail.com.
