# Supplementary material for: Pharmacogenomic profiling of the South Korean population: Insights and implications for personalized medicine
Source: Front Pharmacol. 2024 Dec 3;15:1476765. doi: 10.3389/fphar.2024.1476765 (PMC11650365; doi:10.3389/fphar.2024.1476765)
Supplement: Supplementary file 1 [file Table1.PDF]

Variant	rsID	Variant type	Allele frequency										
			SKR	AFR	AMR	EUR	SAS	EAS					
								EAS	CDX	CHB	CHS	JPT	KHV
g.42525086del	rs5030655	Frame Shift Delete	0	0.0008	0.0029	0.0199	0.001	0	0	0	0	0	0
g.42523805C>T	rs28371725	Intron	0.0168	0.0182	0.062	0.0934	0.1217	0.0377	0.0806	0.034	0.0476	0.0048	0.0253
g.42526694G>A	rs1065852	Missense Mutation	0.4464	0.1127	0.1484	0.2018	0.1646	0.5714	0.629	0.6019	0.6143	0.3606	0.6616
g.42525772G>A	rs28371706	Missense Mutation	0	0.2179	0.0086	0.002	0	0	0	0	0	0	0
g.42523610C>T	rs59421388	Missense Mutation	0	0.1074	0.0029	0	0	0	0	0	0	0	0
g.42525035C>T	rs5030865	Missense Mutation	0	0	0	0	0	0.0099	0.0161	0.0049	0	0.0048	0.0253
g.42525035C>A	rs5030865	Missense Mutation	0										
g.42524947C>T	rs3892097	Intron	0	0.0605	0.1297	0.1859	0.1094	0.002	0	0.0049	0	0	0.0051
g.42524203_42524206del	rs72549351	Frame Shift Delete	0										
g.42523843C>G	rs72549349	Intron	0										
g.42523592G>A	rs147960066	Nonsense Mutation	0	0.000008	0	0	0	0	0	0	0	0	0
g.42522613G>C	rs1135840	Missense Mutation	0.3542	0.5977	0.3284	0.4178	0.3969	0.6401	0.7089	0.7126	0.6897	0.3418	0.7317
g.42526670C>T	rs5030862	Missense Mutation	0										
g.42525912C>G	rs201377835	Intron	0	0.08	0	0	0	0	0	0	0	0	0
g.42524178_42524180del		Frame Shift Delete	0										

Supplementary Table S1. Allele frequency in CYP2D6 across global populations and subgroups of East Asians. SKR, South Korean (our study population); AFR, Africans; AMR, Admixed Americans; SAS, South Asians; EUR, Europeans; EAS, East Asians; CHS, Southern Han Chinese; JPT, Japanese; CHB, Han Chinese; KHV, Kinh Vietnamese; CDX, Dai Chinese.
