# Supplementary material for: Pharmacogenomic profiling of the South Korean population: Insights and implications for personalized medicine
Source: Front Pharmacol. 2024 Dec 3;15:1476765. doi: 10.3389/fphar.2024.1476765 (PMC11650365; doi:10.3389/fphar.2024.1476765)
Supplement: Supplementary file 3 [file Table2.PDF]

Locus	Ranked	Population											
		Allele	Frequency	EAS						AFR*	AMIND*	EUR*	SCSEAI*
				KOR*	CHB*	JPC*	CHS*	CDX*	KHV*				
HLA-A	1	A*02:01	0.15316	0.16500	0.15800	0.11500	0.02000	0.01600	0.02100	0.11459	0.27764	0.27551	0.05784
	2	A*24:02	0.15190	0.21700	0.10500	0.37900	0.22200	0.08100	0.13800	0.02201	0.12920	0.08465	0.13802
	3	A*33:03	0.12785	0.16300	0.06200	0.09700	0.04000	0.10100	0.11500	0.05233	0.00389	0.00321	0.09478
	4	A*11:01	0.10380	0.10800	0.20200	0.08200	0.30300	0.39100	0.22900	0.01119	0.04878	0.06089	0.17533
	5	A*02:06	0.07722	0.07100	0.04700	0.07700	-	0.00800	0.04700	0.00033	0.02745	0.00182	0.01888
	6	A*31:01	0.06962	0.05400	0.01900	0.07100	-	0.00800	0.02100	0.00790	0.07365	0.02698	0.02594
	7	A*26:01	0.03418	0.06000	0.02700	0.08100	0.01000	0.02000	0.02100	0.01243	0.01988	0.03088	0.03871
	8	A*02:07	0.02911	0.03000	0.07700	0.02200	-	0.18500	0.08500	0.00000	0.00003	0.00004	0.02197
	9	A*30:01	0.01899	0.03500	0.05000	0.00100	-	0.00400	0.00300	0.06853	0.01092	0.01299	0.01691
	10	A*03:01	0.01519	0.01800	0.02900	0.00400	-	0.00400	-	0.07834	0.10437	0.13987	0.04987
HLA-B	1	B*44:03	0.09241	0.08500	0.02600	0.08700	-	-	0.03800	0.04243	0.03777	0.04668	0.06086
	2	B*15:01	0.08861	0.10500	0.07000	0.08700	0.03000	0.00800	0.04100	0.00662	0.06161	0.06063	0.01607
	3	B*35:01	0.07595	0.05700	0.03000	0.07600	-	0.00800	0.00300	0.06675	0.09792	0.05596	0.04760
	4	B*51:01	0.07342	0.08400	0.05400	0.07700	0.01500	0.05600	0.03500	0.02409	0.06781	0.04732	0.06697
	5	B*58:01	0.06582	0.06500	0.06000	0.00400	0.17000	0.07700	0.06500	0.04247	0.00699	0.00726	0.04778
	6	B*40:02	0.04684	0.03800	0.01100	0.08600	0.00500	-	0.01200	0.00273	0.03099	0.01263	0.00692
	7	B*46:01	0.04430	0.04400	0.09600	0.03600	0.10000	0.25400	0.11500	0.00007	0.00014	0.00008	0.03405
	8	B*52:01	0.03797	0.02800	0.01700	0.10700	0.02500	0.02800	0.00300	0.01718	0.00656	0.00886	0.05916
	9	B*07:02	0.03544	0.03500	0.03400	0.06500	-	-	-	0.06887	0.10054	0.13061	0.03335
	10	B*54:01	0.03418	0.05900	0.03000	0.07700	0.01500	-	0.02400	0.00000	0.00000	0.00004	0.00381

* : Gonzalez-Galarza, Faviel F., et al. "Allele frequency net database (AFND) 2020 update: gold-standard data classification, open access genotype data and new query tools." *Nucleic acids research* 48.D1 (2020): D783-D788.

- : No information on alleles.

Supplementary Table S2. Comparison between South Korean population and other populations. KOR, South Korea (n=485); CHB, China Beijing Shijiazhuang Tianjian Han (n=618); JPC, Japanese in Central (n=371); CHS, Southern Han Chinese (n=100); CDX, Chinese Dai in Southwest (n=124); KHV, Kinh in Ho Chi Minh City (n=170); AFR, African (USA NMDP African, n=28557); AMIND, North American Indian(USA NMDP North American Amerindian, n=35791); EUR, European(USA NMDP European Caucasian, n=1242890); SCSEAI, Southeast Asian (USA NMDP Southeast Asian, n=27978)
