# Supplementary material for: Pharmacogenomic profiling of the South Korean population: Insights and implications for personalized medicine
Source: Front Pharmacol. 2024 Dec 3;15:1476765. doi: 10.3389/fphar.2024.1476765 (PMC11650365; doi:10.3389/fphar.2024.1476765)
Supplement: Supplementary file 4 [file Image2.pdf]

A

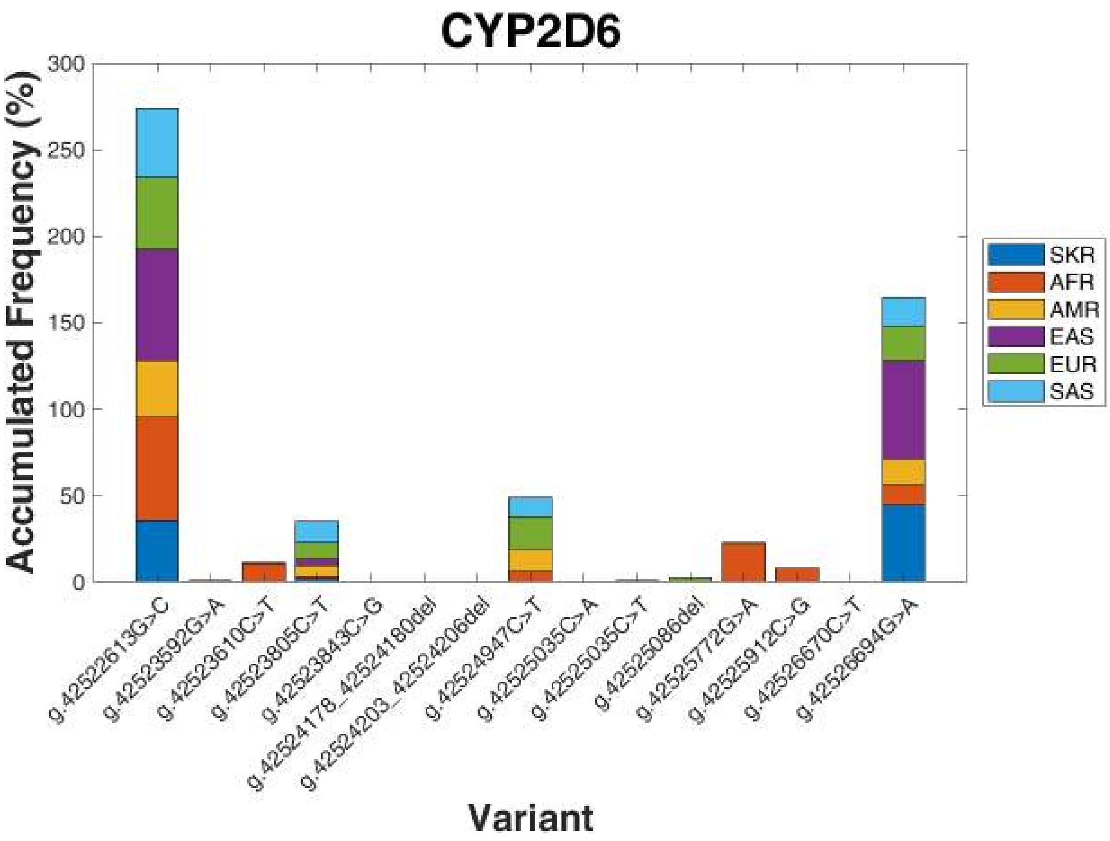
CYP2D6
Accumulated Frequency (%)
300
250
200
150
100
50
0
SKR
AFR
AMR
EAS
EUR
SAS
g.42522613G>C
g.42523592G>A
g.42523610C>T
g.42523805C>T
g.42523843C>G
g.42524178_42524180del
g.42524203_42524206del
g.42524947C>T
g.42525035C>A
g.42525035C>T
g.42525086del
g.42525772G>A
g.42525912C>G
g.42526670C>T
g.42526694G>A
Variant

B

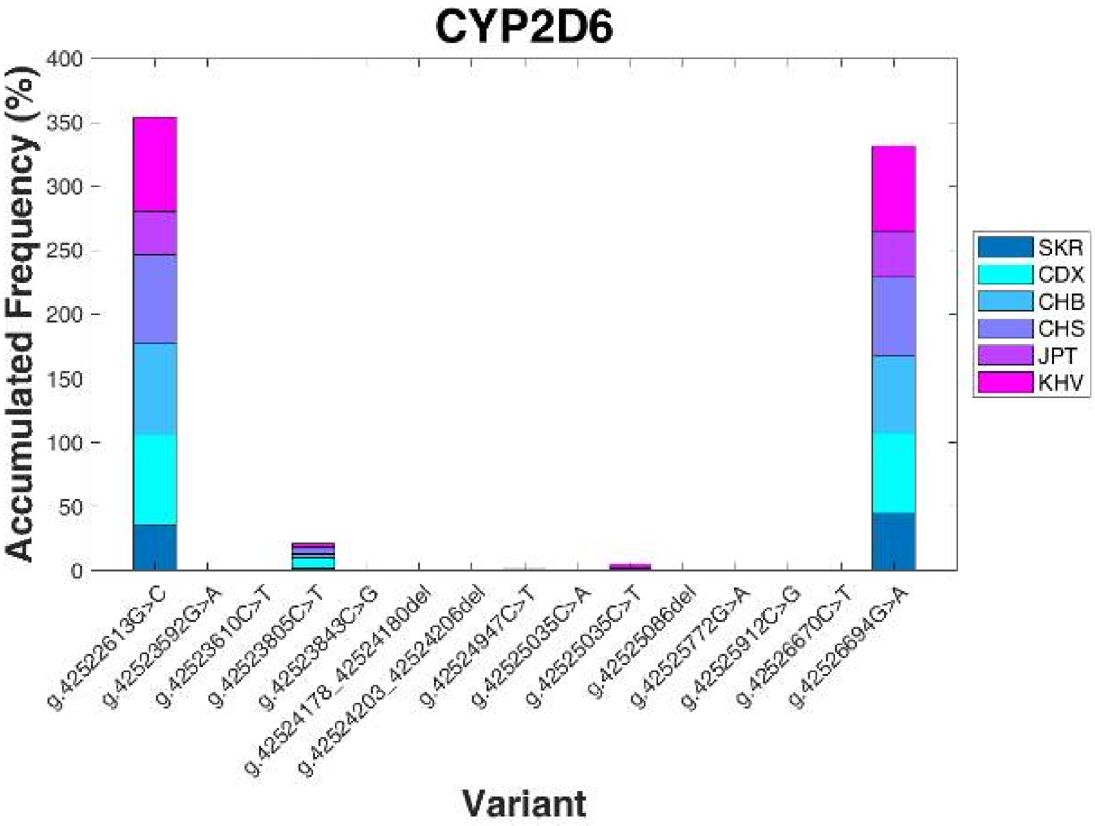

Supplementary Figure S2. Frequency comparison of CYP2D6 variants in our study population versus the 1KG population. (a) Cumulative frequency of variants by ethnicity in the CYP2D6 gene. (b) Cumulative frequency of variants within East Asian (EAS) ethnicities in the CYP2D6 gene. The x-axis represents the observed types of variants for the CYP2D6 gene, while the y-axis represents the corresponding cumulative percentages. In these graphs, distinct colors denote different populations. SKR, South Korean (our study population); AFR, Africans; AMR, Admixed Americans; SAS, South Asians; EUR, Europeans; EAS, East Asians; CHS, Southern Han Chinese; JPT, Japanese; CHB, Han Chinese; KHV, Kinh Vietnamese; CDX, Dai Chinese.
