# Supplementary material for: Pharmacogenomic profiling of the South Korean population: Insights and implications for personalized medicine
Source: Front Pharmacol. 2024 Dec 3;15:1476765. doi: 10.3389/fphar.2024.1476765 (PMC11650365; doi:10.3389/fphar.2024.1476765)
Supplement: Supplementary file 6 [file Table3.PDF]

Drug : Rosuvastatin						
**Phenotype**	**ABCG2 Normal function**		**ABCG2 Decreased function**		**ABCG2 Poor function**	
**SLCO1B1 Increased function**	Standard	0	Standard	0	Down	0
**SLCO1B1 Normal function**	Standard	174	Standard	103	Down	23
**SLCO1B1 Decreased / SLCO1B1 Possible Decreased function**	Standard	0	Standard	0	Down	0
**SLCO1B1 Poor function**	Down	50	Down	34	Down	5
**SLCO1B1 Unknown function**	Unknown	2	Unknown	5	Unknown	0
**Drug : Fluvastatin**						
**Phenotype**	**CYP2C9 Normal metabolizer**		**CYP2C9 Intermediate metabolizer**		**CYP2C9 Poor metabolizer**	
**SLCO1B1 Increased function**	Standard	0	Down	0	Down	0
**SLCO1B1 Normal function**	Standard	269	Down	30	Down	1
**SLCO1B1 Decreased / SLCO1B1 Possible Decreased function**	Standard	0	Down	0	Alternative	0
**SLCO1B1 Poor function**	Down	82	Alternative	7	Alternative	0
**SLCO1B1 Unknown function**	Unknown	6	Unknown	1	Unknown	0
**Drug : Mercaptopurine, Azathioprine, Thioguanine**						
**Phenotype**	**TPMT Normal metabolizer**		**TPMT Intermediate metabolizer**		**TPMT Poor metabolizer**	
**NUDT15 Normal metabolizer**	Standard	298	Down	8	Down	1
**NUDT15 Intermediate metabolizer**	Down	65	Down	6	Down	0
**NUDT15 Poor metabolizer**	Down	3	Down	0	Down	0
**NUDT15 Unknown**	Unknown	15	Unknown	0	Unknown	0
**Drug : Phenytoin**						
**Phenotype**	**CYP2C9 Normal metabolizer**		**CYP2C9 Intermediate metabolizer**		**CYP2C9 Poor metabolizer**	
**HLA-B*15:02 Positive**	Alternative	0	Alternative	0	Alternative	0

**HLA-B*15:02 Negative**	Standard	356	Down	38	Down	1
**Drug : Carbamazepine**						
**Phenotype**	**HLA-A*31:01 Positive**		**HLA-A*31:01 Negative**			
**HLA-B*15:02 Positive**	Alternative	0	Alternative	0		
**HLA-B*15:02 Negative**	Alternative	53	Standard	342		

Supplementary Table S3. Count of drug recommendations by multiple phenotype combinations for each drug in South Korean population.
