# Supplementary material for: Pharmacogenomic profiling of the South Korean population: Insights and implications for personalized medicine
Source: Front Pharmacol. 2024 Dec 3;15:1476765. doi: 10.3389/fphar.2024.1476765 (PMC11650365; doi:10.3389/fphar.2024.1476765)
Supplement: Supplementary file 12 [file Image1.pdf]

A

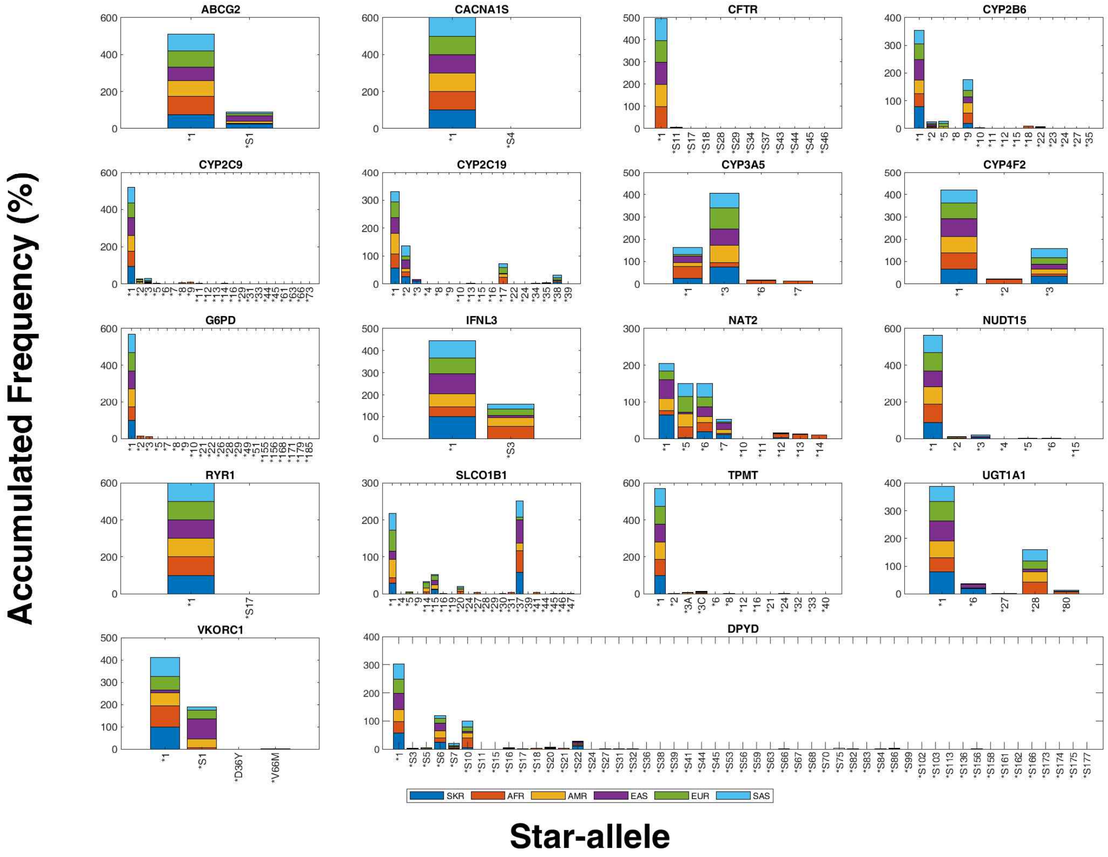
ABCG2
CACNA1S
CFTR
CYP2B6
CYP2C9
CYP2C19
CYP3A5
CYP4F2
G6PD
IFNL3
NAT2
NUDT15
RYR1
SLCO1B1
TPMT
UGT1A1
VKORC1
DPYD
SKR
AFR
AMR
EAS
EUR
SAS
Accumulated Frequency (%)
Star-allele

B

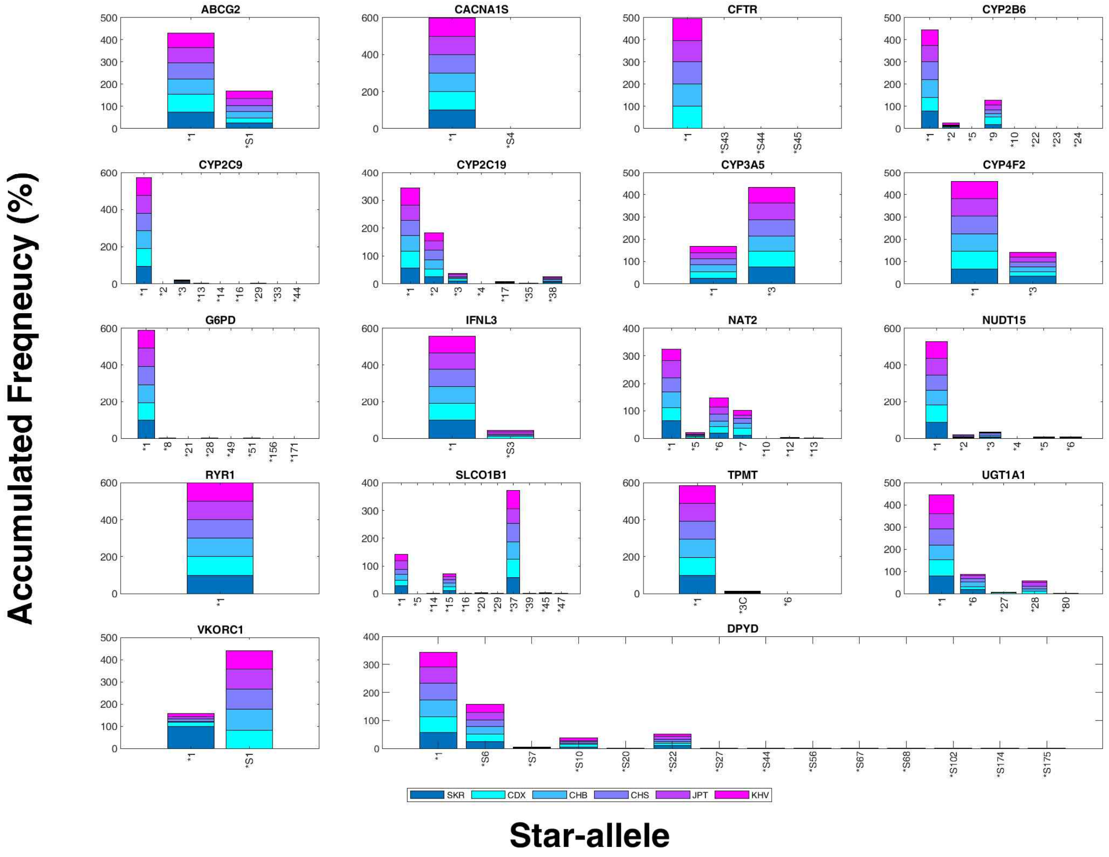

Supplementary Figure S1. Comparison of Star-Allele Frequencies Between Our Study Population and the 1000 Genomes Project (1KG) Populations. (a) Cumulative frequency of star-alleles by ethnicity in the core pharmacogenes. (b) Cumulative frequency of star-alleles within East Asian (EAS) ethnicities in the core pharmacogenes. The x-axis represents the observed types of star-alleles for each gene, while the y-axis represents their corresponding cumulative percentages. In these graphs, distinct colors denote different populations. Unique colors correspond to each distinct predicted phenotype in these graphs. SKR, South Korean (our study population); AFR, Africans; AMR, Admixed Americans; SAS, South Asians; EUR, Europeans; EAS, East Asians; CHS, Southern Han Chinese; JPT, Japanese; CHB, Han Chinese; KHV, Kinh Vietnamese; CDX, Dai Chinese.
